# Metal ion dyshomeostasis in neurological disorders: from pathological mechanisms to targeted neuroprotective and regenerative strategies

**DOI:** 10.3389/fphar.2026.1916973

**Published:** 2026-08-06

**Authors:** Mengqiang Yao, Chunlei Xing, Xin Li, Xiaochen Sun, Chenxi Zhang, Xingji You, Li Su

**Affiliations:** 1 Institute of Translational Medicine, Shanghai University, Shanghai, China; 2 School of Medicine, Shanghai University, Shanghai, China

**Keywords:** acquired brain injury, chelating agents, metal ions, neurodegenerative diseases, oxidative stress

## Abstract

Metal ions play an indispensable role in maintaining the physiological functions of the nervous system, while their dyshomeostasis also drives the pathogenesis of various neurological disorders. Their involvement spans from acute traumatic brain injury to chronic neurodegenerative diseases, in which they participate in pathological regulation through mechanisms such as homeostatic disruption, oxidative stress, induction of specific cell death pathways, thereby influencing secondary neuronal death and impaired regeneration. This review summarizes the pathophysiological roles of metal ions and their associations with acquired brain injury and neurodegenerative diseases, aiming to further elucidate targetable therapeutic windows for neuroprotection and neural repair. Furthermore, we evaluate the translational challenges of clinical-stage metal chelators (e.g., deferiprone, PBT2) and highlight novel nano-delivery strategies designed to cross the blood-brain barrier to reverse subcellular metal toxicity. This provides a clear therapeutic roadmap for developing next-generation neuroprotective drugs.

## 1 Introduction

The central nervous system (CNS) maintains a strict and compartmentalized homeostasis of metal ions. This is fundamentally different from the peripheral tissues. Essential trace metals like iron (Fe), copper (Cu), zinc (Zn), and manganese (Mn) are indispensable cofactors in the brain for neurotransmitter synthesis, synaptic plasticity, and myelin lipid biosynthesis. However, because these transition metals are highly redox-active, the brain requires a specialized network of cells to prevent abnormal metal accumulation and the oxidative damage it causes. At the macroscopic level of the organism, the blood-brain barrier (BBB) plays the primary role as a selective gatekeeper, extremely strictly restricting the infiltration of peripheral transition metals into the brain parenchyma through the expression of specific transporters such as transferrin receptor (TfR) and copper transporter 1 (Ctr1) (Gao et al., 2023).

**FIGURE 1:**
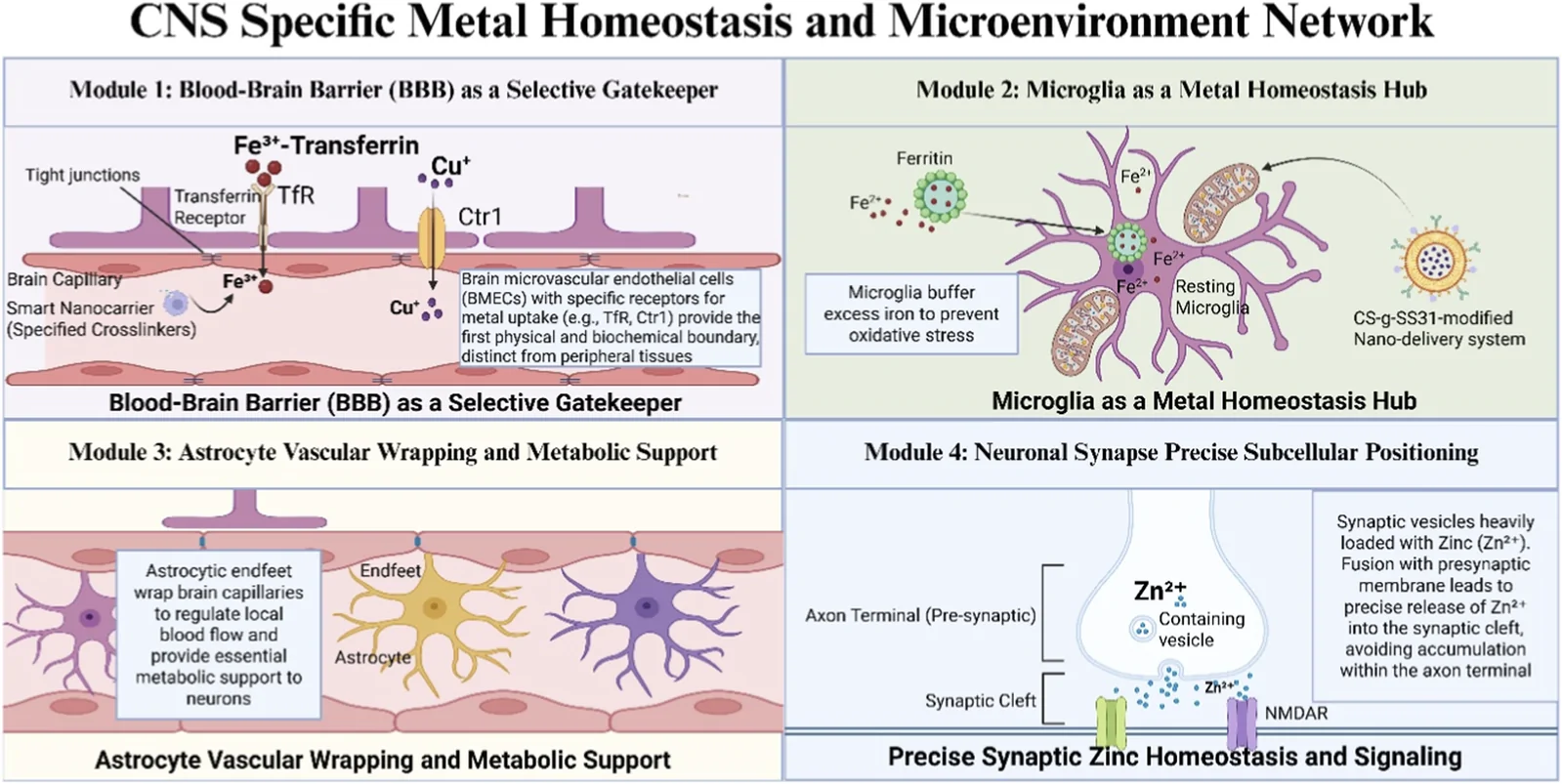
Regional regulatory networks governing CNS-specific metal ion homeostasis. (Module 1) The BBB acts as a selective gatekeeper: The brain microvascular endothelial cells strictly restrict the trans-capillary infiltration of transition metals through specific receptors (such as TfR that binds Fe^3+^-transthyretin, and Ctr1 that transports Cu^+^). Introducing intelligent nanocarriers functionalized with specific cross-linkers aims to overcome this physical barrier. (Module 2) Microglia as Metabolic Buffer Hubs: At rest, microglia suppress redox-driven oxidative stress by actively taking up extracellular free Fe^2+^ and safely sequestering it within ferritin complexes; the introduction of mitochondria-targeted modifications (such as CS-g-SS31 grafting) into nanodelivery systems allows for precise targeting of these metabolic hubs. (Module 3) Astrocyte vascular wrapping and metabolic support: Astrocyte end-feet tightly wrap around the basal membrane of brain capillaries, working together within the glial limitans network to regulate local blood flow and provide essential metabolic support. (Module 4) Zinc dynamics at the subcellular level in tripartite synapses: Zn^2+^ are highly concentrated in synaptic vesicles under physiological conditions and are directly released into the synaptic cleft during vesicle fusion, effectively preventing abnormal accumulation within the presynaptic axonal terminal and finely modulating the excitability of the postsynaptic membrane’s NMDAR.

In the brain’s tissue microenvironment, the glial cell network acts as a key metabolic hub, dynamically buffering fluctuations of extracellular metals to protect vulnerable neurons. As resident immune cells of the central nervous system, microglia not only play a role in regulating neuroinflammation but also serve as key fine-regulators of iron metabolism in the brain; They actively take up excess free iron outside the cells and safely store it in ferritin complexes, thereby stopping the neurotoxic cascade driven by the Fenton reaction right from the source (Nnah and Wessling-Resnick, 2018). Meanwhile, the foot processes of astrocytes closely surround the brain capillaries, functioning to regulate local blood flow and providing essential metabolic support for neurons. At the microscopic subcellular scale, the kinetic distribution of metal ions is also subject to extremely precise positioning. For instance, in a physiological state, zinc ions are highly concentrated in the presynaptic vesicles and are directly released into the synaptic cleft upon stimulation - rather than accumulating within the presynaptic axonal terminal - thereby finely modulating the excitability of the postsynaptic receptors (Krall et al., 2021). Disruption of this intricate and tightly interconnected cellular network is a key upstream catalyst for redox-driven metal toxicity in acquired brain injury and chronic neurodegenerative diseases. So, this review systematically explains the pathophysiological roles of these metal ions and clarifies identifiable therapeutic windows, aiming to provide a clear pharmacological roadmap for developing the next-generation of neuroprotective and regenerative interventions (Figure 1).

## 2 The effects of metal ions on acquired brain injury (ABI)

**FIGURE 2:**
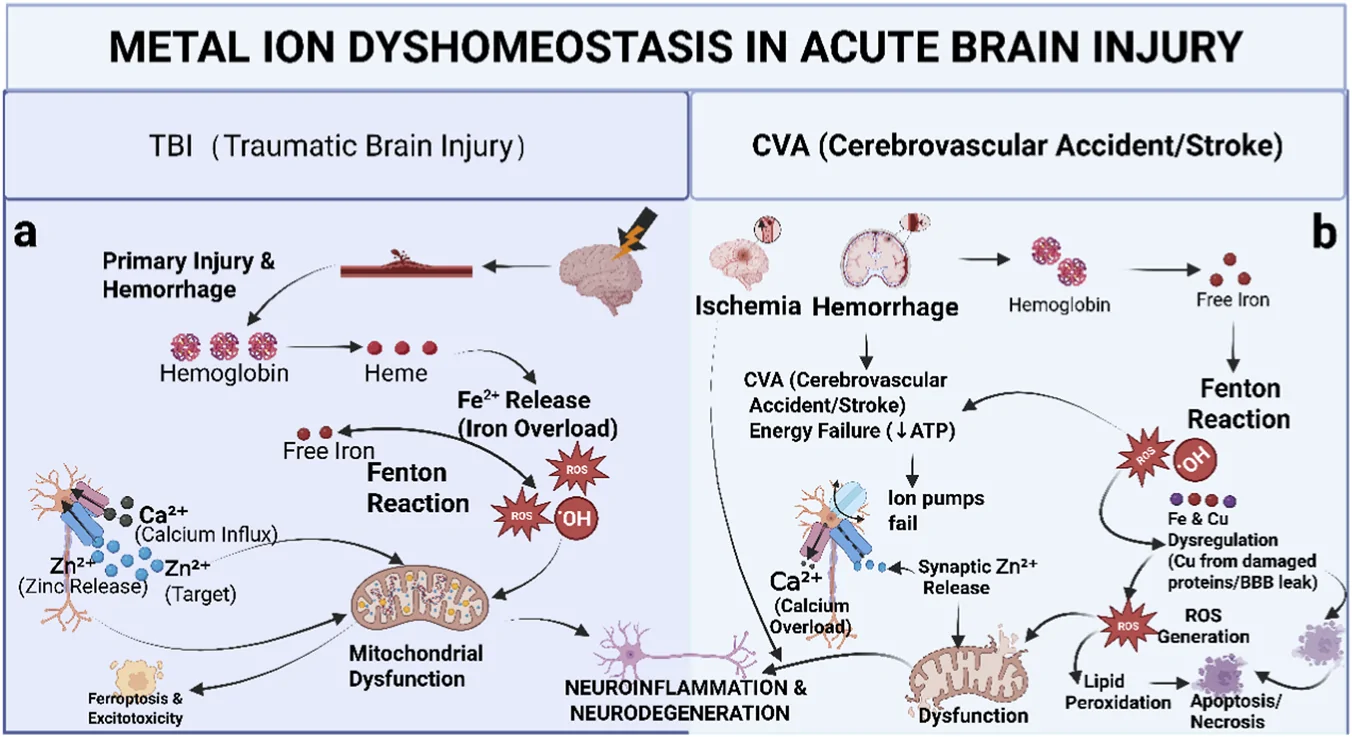
Metal ions and acquired brain injury. **(a)** Metal ions and traumatic brain injury. Following TBI, a series of reactions promote the release of Fe^2+^, leading to iron overload and triggering the Fenton reaction, which generates ROS and hydroxyl radicals (·OH), thereby impairing mitochondrial energy production. Excitatory neurotransmitters such as glutamate, when over-activating receptors, can cause calcium influx. Zinc ions within the cells are released from storage sites (such as mitochondria) as a key toxic mediator, further exacerbating cell damage. Eventually, excitotoxicity is induced. **(b)** Metal Ions and Stroke. Ischemic stroke occurs due to the depletion of energy in the brain tissue (ATP decline), which triggers the dysfunction of ion pumps, resulting in intracellular calcium overload and the release of zinc ions at synapses, and damaging mitochondrial function. In hemorrhagic stroke, the decomposition of hemoglobin released by red blood cells generates a large amount of free iron. CVA ultimately triggers the Fenton reaction, leading to lipid peroxidation and oxidative stress. Severe oxidative stress, calcium overload, and mitochondrial dysfunction collectively trigger neuronal apoptosis and necrosis, which in turn induce an inflammatory response, ultimately resulting in irreversible neurodegeneration and brain dysfunction.

ABI is caused by various diseases or injuries during or after birth that result in changes in brain function. Among them, traumatic brain injury (TBI) and stroke (CVA) are the most common causes (Brazinova et al., 2021; Goldman et al., 2022). Here, we mainly introduce the effects of metal ions on TBI and CVA (Figure 2).

### 2.1 Metal ions and TBI

The research has found that one of the main symptoms after TBI occurs is cognitive impairment (Skandsen et al., 2010). Furthermore, long-term cognitive impairment may lead to neurodegenerative diseases such as Alzheimer’s disease (AD) (Van Den Heuvel et al., 2007), Amyotrophic Lateral Sclerosis (ALS) (Chen et al., 2007), and Parkinson’s disease (PD) (Factor and Weiner, 1991). Metal zinc (Zn) is generally regarded as a neurotrophic metal (Wang B. et al., 2023). Following a TBI, the massive release of zinc ions from presynaptic vesicles leads to a specific depletion of zinc in the presynaptic membrane; at the same time, these free zinc ions flood into and accumulate within the damaged postsynaptic neuron. This classic “zinc translocation” mechanism was first revealed in epilepsy models (Frederickson et al., 1989). This was subsequently confirmed to be a key trigger for secondary neuronal death following TBI (Suh et al., 2000). The fluctuations in the damaged area Zn may trigger neurotoxicity, thereby exacerbating brain damage. Additionally, the fluctuations in Zn may be related to the occurrence of cognitive impairment after TBI (Frederickson et al., 2004). The neurotoxicity of Zn primarily causes neuronal damage through three mechanisms: increased production of reactive oxygen species (ROS), activation of the mitogen-activated protein kinase (MAPK) signaling pathway, regulation of potassium channels and apoptosis, and mitochondrial damage leading to energy depletion and further oxidative damage (Krall et al., 2021). The research found that the content of iron (Fe) in the brain increased sharply after TBI occurred, indicating a close relationship between Fe and TBI (Liu et al., 2013). Ferritin is responsible for iron storage in the brain; a complete ferritin molecule is a polymeric complex composed of 24 subunits, consisting of heavy chains (H-chains) and light chains (L-chains) (Connor et al., 2001). In the iron homeostasis regulation, the H chain of ferritin is responsible for the efficient turnover and utilization of iron, while the L chain specifically mediates the stable precipitation and long-term storage of iron. Research has shown that the number of Fe-positive cells around the damaged cortex in TBI significantly increases, and they are mainly found in glial cells. The increase in Fe after TBI may be related to the intracranial hemorrhage and the destruction of the BBB caused by TBI, which leads to the release of heme containing iron; subsequently, the free heme is cleaved and metabolized by HO-1 into carbon monoxide (CO), biliverdin, and free iron (Fe) (Liu et al., 2013). Excessive iron can trigger the Fenton reaction, leading to oxidative stress and lipid peroxidation, which ultimately result in neuronal damage (Liu J. et al., 2022). The ferritin H chain can store excess iron, which may represent a protective response; however, following TBI, the expression of the ferritin H chain in neurons is significantly upregulated, which may have toxic effects on the nervous system (Liu et al., 2013). Studies have shown that after TBI occurs, Cu levels were significantly elevated in the damaged area, and this abnormal accumulation further aggravates the synaptic damage after TBI, the hint is that Cu affects the pathological development of TBI (Chang et al., 2025).

TBI often leads to increased intracranial pressure (ICP), which is the primary cause of death in TBI cases. By measuring the levels of serum copper (Cu) and ceruloplasmin in patients, it is possible to predict the changes in intracranial pressure after TBI. This suggests that peripheral blood copper levels may serve as an early biomarker for predicting the risk of developing elevated intracranial pressure following TBI (Dash et al., 2010). Cu is indispensable for mitochondrial respiration and as a cofactor of antioxidant enzymes (An et al., 2022). Copper affects wound healing and tissue regeneration, a finding consistent with the observation of copper accumulation above physiological baseline levels in damaged local tissues (Mirastschijski et al., 2013). This spatial redistribution of metal ions also occurs in TBI. Through isotope tracing (PET/CT), it was discovered that the local neuroinflammation induced by TBI significantly promotes the abnormal uptake of copper in the cortical injury area, resulting in an abnormal increase in copper content in this area (Peng et al., 2015). This localized redistribution of copper at the site of injury may serve as a compensatory response to tissue self-repair, but it may also trigger secondary neurotoxicity due to copper overload. Copper (Cu) is a key element in maintaining the homeostasis of wound healing, while zinc (Zn) and selenium (Se) drive the tissue repair process through synergistic interactions with copper. Although direct research evidence supporting the specific effects of Se in the pathophysiology of TBI is currently lacking, it is hypothesized that Se may have a potential association with TBI based on its interactions with Cu and its role in promoting tissue repair (Mirastschijski et al., 2013).

### 2.2 Metal ions and cerebrovascular accident (CVA)

CVA, commonly known as a stroke, primarily includes ischemic stroke and hemorrhagic stroke. Ischemic stroke results from impaired blood flow to local tissues due to vascular occlusion, leading to an interruption in the supply of metabolic substrates and causing ischemic necrosis of the affected tissues and neurons (Feske, 2021). Hemorrhagic stroke, primarily intracerebral hemorrhage (ICH), includes two subtypes: intracerebral hemorrhage and subarachnoid hemorrhage, and it is the most lethal form of stroke (Ohashi et al., 2023).

Following an acute ischemic stroke (AIS), neuroinflammation develops rapidly and persists for several days; simultaneously, inflammatory markers are elevated in ischemic stroke. Therefore, neuroinflammation is a key process following both AIS and ICH (Alsbrook et al., 2023). Copper (Cu) is a key component in many critical metabolic processes of brain cells and in the formation of neurotransmitters (Nam et al., 2020). After the AIS event, the copper homeostasis in the brain undergoes significant changes, causing it to be in an imbalanced state (Burkhead et al., 2009). At the same time, studies have found that during a stroke, the homeostasis of intracellular copper ion uptake and efflux becomes disrupted due to impaired ATP production, and the accumulation of copper ions within cells may lead to copper-mediated cell death (Wang et al., 2022a). Cu not only can induce cell death through copper-mediated apoptosis during stroke, but also can trigger cell death by increasing zinc (Zn) levels within the cells (Tanaka et al., 2018). Therefore, targeting a reduction in Cu/Zn levels at the stroke site can improve stroke outcomes (Qi et al., 2023; Peng et al., 2024). Mitochondria are the core cellular organelles responsible for the synthesis of adenosine triphosphate (ATP) within cells. They primarily convert nutrients into biochemical energy through the oxidative phosphorylation process. Cu is closely related to mitochondria and cells that are more sensitive to ATP are also more sensitive to copper. The brain is a high-oxygen-consuming organ, so it is more sensitive to copper than cells in other organs (Uzdensky, 2020; Tsvetkov et al., 2022). Since copper-mediated cell death occurs in mitochondria, targeting Cu/inhibiting cuproptosis has a positive effect on the treatment of stroke (Wang et al., 2022a).

Unlike the ischemic cascade reaction, ICH represents a key pathological paradigm where excessive transition metals act as the absolute core executor of secondary brain injury. Following an acute cerebral hemorrhage, blood instantly leaks into the brain parenchyma, ultimately leading to massive hemolysis of red blood cells. The breakdown of these physical and biochemical barriers releases excess hemoglobin, which is quickly broken down into free heme. Subsequently, the inducible heme oxygenase-1 (HO-1) breaks down heme into biliverdin, CO and Fe^2+^. Although the upregulation of HO-1 is initially a protective response to clear heme, its excessive activation can rapidly exceed the buffering capacity of endogenous ferritin complexes, leading to a catastrophic expansion of the intracellular labile iron pool (LIP). These unchelated Fe^2+^ directly catalyze the Fenton reaction, producing highly toxic hydroxyl radicals (^.^OH) and lipid peroxides, which specifically drive neurons to undergo ferroptosis (Shi et al., 2024). Therefore, inhibiting this localized iron overload and suppressing the excessive activation mediated by HO-1 has become the core focus of modern research on neuroprotection for cerebral hemorrhage. In addition to the classic iron-dependent ferroptosis, recent cutting-edge evidence suggests that copper homeostasis imbalance and mitochondrial copper-mediated cell death are also critical, concurrently occurring factors in the ICH. As red blood cells break down, a lot of copper-containing proteins (like plasma ceruloplasmin) leak into the bleeding microenvironment, worsening the structural damage to the BBB and causing localized copper buildup. In the hypoxic ischemic zone of cerebral hemorrhage with high oxidative stress, excessive copper ions flood into the cytoplasm of adjacent neurons and glial cells through the upregulated Ctr1. Once inside the mitochondria, these copper ions bind specifically to acyl-modifying enzymes in the tricarboxylic acid (TCA) cycle, causing them to form toxic, insoluble abnormal aggregates. This molecular aggregation blocks downstream bioenergetic coupling, causing acute mitochondrial dysfunction and actively triggering cuproptosis (Zhu et al., 2024). Therefore, the hemorrhagic penumbra is a highly complex dual-transition-metal core target area where ferroptosis and cuproptosis coexist. This implies that future intervention strategies must move towards “dual-metal combined targeting” in order to maximize the neuroprotective efficacy after cerebral hemorrhage.

Copper-zinc superoxide dismutase (CuZn-SOD) is present in the cytoplasmic matrix, the inner mitochondrial membrane, and the cell nucleus. It can regulate the production of harmful substances ROS in cells and is crucial for regulating redox in the CNS (Crapo et al., 1992; Wood and Thiele, 2009). Copper is a key cofactor for lysyl oxidase; by promoting the covalent cross-linking of elastin and collagen, it helps maintain the elasticity and toughness of blood vessel walls. Studies have shown that copper deficiency impairs cholesterol degradation, leading to its deposition in blood vessel walls and the development of atherosclerosis (Klevay, 2000). However, a significant increase in serum Cu was found in patients with ischemic stroke. It is speculated that this may be related to the increased release of copper from acute atherosclerosis, possibly to the activation of catecholamine synthase due to sympathetic nerve activation, and possibly to the positive correlation between serum copper and very low-density lipoprotein. Therefore, it can be inferred that serum copper affects the course of ischemic stroke (Zhang et al., 2020).

Iron (Fe) may be involved in the release of free radicals, excitotoxicity and neuroinflammatory responses in ischemic stroke (Nagy and Nardai, 2017). Ischemic stroke leads to structural disruption of the BBB, thereby compromising its physiological role in isolating the CNS from the peripheral circulation. This results in abnormal infiltration of peripheral blood-derived iron (Fe) into the brain parenchyma, which in turn exacerbates secondary brain injury; simultaneously, the depletion of peripheral iron also aggravates ischemic brain injury (Guo et al., 2023). Current evidence indicates that Fe promotes neuronal death in ischemic stroke primarily through ferroptosis, and abnormal iron metabolism with cerebral iron accumulation exacerbates brain injury. Following ischemic stroke, TfR is upregulated, facilitating Fe uptake and exacerbating oxidative damage; NCOA4 regulates ferritinophagy, promoting ferroptosis; and Tau protein deficiency impairs Fe efflux from the CNS, leading to Fe accumulation and neuronal injury. Additionally, ischemic stroke impairs glutathione (GSH) synthesis, diminishing antioxidant capacity, and disrupts lipid metabolism, generating lipid peroxides that induce ferroptosis. Endoplasmic reticulum (ER) stress following ischemic stroke also promotes ferroptosis, which in turn exacerbates ER stress, establishing a vicious cycle (Li et al., 2024). Studies have shown differential activities of CuZn-SOD and manganese superoxide dismutase (Mn-SOD) in patients with ischemic stroke, with cerebral CuZn-SOD activity being higher than that in controls, whereas Mn SOD activity is significantly lower, suggesting a potential role for manganese (Mn) in ischemic stroke (Imai and Okabe, 2011). Manganese homeostasis imbalance is relatively common in patients with hereditary hemorrhagic telangiectasia (HHT). Given that HHT patients are highly susceptible to abnormal embolic ischemic strokes, it has been suggested that severe manganese homeostasis imbalance (abnormal deposition in the basal ganglia) may serve as an important biomarker indicating the presence of potentially high-risk vascular malformations associated with stroke (Tang Q. et al., 2024). Manganese is associated with neurotoxicity. Long-term exposure to manganese may cause neurotoxicity and lead to stroke. Studies have shown that a worker who had long-term exposure to manganese exhibited symptoms similar to a stroke. After treatment and enhanced personal protective measures at work, it was found that the symptoms of the stroke-like condition significantly decreased (Alikunju et al., 2023). Zn, as a crucial neuroregulatory factor, plays an indispensable role in ensuring normal signal transmission in the brain and the stability of the nervous system. After ischemic stroke occurs, the imbalance of Zn in microglia and astrocytes leads to cytotoxicity. Meanwhile, Zn can accumulate in hippocampal neurons through the zinc transport system or zinc-binding proteins on the cell membrane. This uncontrolled intracellular zinc load induces neuronal cell death. This uncontrolled state triggers upstream pathways such as MAPK, which leads to abnormal phosphorylation of serine/threonine kinase CDK5 at position 15 (Tyr15). This modification breaks the conventional regulatory mechanism of CDK5, causing its kinase activity to be abnormally activated, and ultimately mediates neuronal death in ischemic injury (Tuo et al., 2018; Higashi et al., 2019). Current research shows that increasing the intake of Zn in daily diet can help prevent or reduce stroke and related symptoms (Huang et al., 2024). Meanwhile, insufficient daily zinc intake can lead to abnormal brain function and may trigger a hemorrhagic stroke (Grüngreiff et al., 2020).

Se participates in the regulation of nervous system homeostasis. Selenium nanoparticles possess a variety of neuroprotective properties, with mechanisms involving the scavenging of ROS, the inhibition of apoptotic cascades, and the maintenance of endoplasmic reticulum and calcium ion homeostasis. Additionally, they can enhance the tolerance of brain cells to ischemic stress by regulating glial cell function and neurotrophic signaling (Turovsky et al., 2024). The neuroprotective effect of Se does not merely stem from its role as a trace element supplement, but rather lies in its ability to trigger a set of transcriptional adaptive programs (Transcriptional Adaptive Program) to enhance the resilience of cells. As a core selenoprotein, the selective synthesis of glutathione peroxidase 4 (GPX4) is highly dependent on the body’s selenium level, studies have demonstrated that selenium induces the expression of GPX4 and related defense genes at the transcriptional level by synergistically activating the transcription factors TFAP2c and Specific Protein 1 (Sp1). This process significantly enhances the protective barrier of neurons against ferroptosis stimuli and, by maintaining the activity of lipid peroxide reductases, effectively counteracts the cascade of cytotoxicity triggered by stroke. Therefore, this selenium-driven transcriptional regulatory axis not only reveals the activation pathway of endogenous protective mechanisms but also provides precise potential therapeutic targets for clinical intervention in stroke (Alim et al., 2019). The level of Se in the body affects the prognosis of severe stroke (Liampas et al., 2023). Trivalent chromium (Cr) is involved in glucolipid metabolism and glucose regulation (Anderson, 1997). Studies have shown that stroke can induce hyperglycemia, and dysregulation of tissue chromium levels may contribute to cerebral infarction and hyperglycemia; restoring tissue chromium levels may alleviate post-stroke cerebral infarction and hyperglycemia (Chen et al., 2016). Metal implants are commonly used in orthopedic treatment, often containing Cr. Research has demonstrated a positive correlation between a history of metal implants and increased stroke risk, suggesting that slow release of implant-derived metal ions may be a potential risk factor for cerebrovascular events (Wu et al., 2024).

### 2.3 Metal ions and combined pathologies in acute brain injury

Although iron, copper, and zinc are the primary bioactive metals involved in acute brain injury, recent cutting-edge pharmacological research has shown that the homeostatic regulation of Mn, Se, and Ca also fundamentally determines the survival outcome of the damaged neural microenvironment. Mn is a basic element for nerve signal transmission. However, long-term environmental exposure or hereditary metabolic breakdown can cause it to accumulate acutely in the basal ganglia, leading to severe neurotoxicity that clinically resembles an acute cerebrovascular defect. Interestingly, the differential activity tracking revealed that although CuZn-SOD in the brain would increase after acute stroke to counter the initial oxidative stress, the activity of mitochondrial Mn-SOD experienced a sharp decline. This suggests that preserving the mitochondrial Mn pool is a key therapeutic target for reversing the hyperacute-phase bioenergetic collapse (Imai and Okabe, 2011).

Unlike the toxic cascade reactions involving transition metals, Se, when the brain suffers from ischemic injury, can orchestrate a highly coordinated set of transcriptional adaptive programs to enhance the resilience of neurons. Intracellular selenium flux does not act as a passive chemical scavenger, but rather by synergistically activating the specific transcription factor AP-2c (TFAP2c) and specific protein 1 (Sp1). This specific transcriptional regulatory axis directly boosts the production of GPX4 and the downstream lipid-based antioxidant defense systems, creating a strong molecular barrier that protects cortical and hippocampal neurons from ferroptosis triggered after a stroke (Alim et al., 2019). Meanwhile, selenium nanoparticles have become a leading delivery platform capable of stabilizing endoplasmic reticulum stress and correcting excessive activation of Ca^2+^ (Turovsky et al., 2024). During the acute phase of a brain injury, the collapse of ATP-dependent membrane ion pumps can cause massive, uncontrolled calcium influx into the cytoplasm, disrupt the mitochondrial membrane potential, and activate the deadly calpain pathway. By strategically deploying multifunctional selenium-based nanocatalysts or precisely targeting Ca/Mn homeostatic channels, modern neuropharmacology is able to simultaneously suppress calcium-driven excitotoxicity and ferroptosis cascades, establishing a promising interdisciplinary frontier for precise clinical interventions.

## 3 Microglia: the pathological bridge linking metal dyshomeostasis and neuroinflammation

As the main resident immune sentinels of the CNS, microglia are not just passive bystanders—they’re also key regulators of metal balance in the brain. They are equipped with a robust system for processing biometals—including the TfR, divalent metal transporter 1 (DMT1), and high baseline levels of ferritin. During the early stages of a healthy brain or an acute injury, microglia actively take up excess iron from the extracellular environment and safely store it in ferritin complexes. This metabolic buffering ability is crucial for neutralizing free unstable iron that triggers Fenton reactions and protecting fragile neurons from acute oxidative damage (Nnah and Wessling-Resnick, 2018).

However, this protective isolation is an extremely crucial double-edged sword. Progressive metal overload inevitably leads to the saturation of microglia’s ferritin storage capacity, thereby inducing profound metabolic and transcriptomic reprogramming. Microglia with iron toxicity undergo a phenotypic switch, heading into a hyper-inflammatory state, characterized by the continuous release of pro-inflammatory cytokines (like TNF-α and IL-1β) and neurotoxic ROS. Recent cutting-edge transcriptomics and functional evidence show that when microglial metal balance is not maintained, it can trigger ferroptosis stress in microglia, which directly leads to non-cell-autonomous neuron death and actively breaks down nearby synaptic structures (Liddell et al., 2024).

**FIGURE 3:**
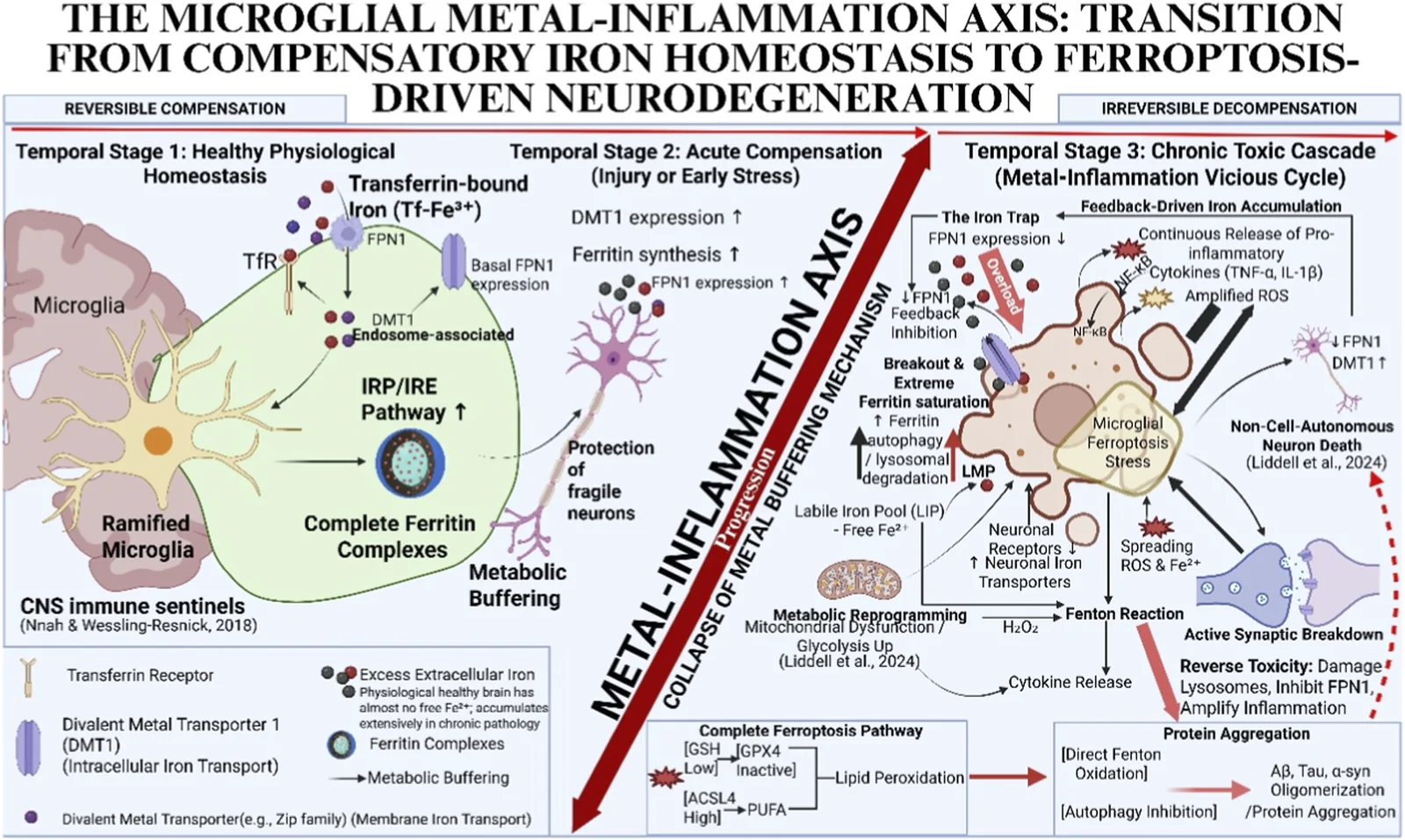
The microglial metal-inflammation axis: The transition from compensatory iron homeostasis to ferroptosis-driven neurodegenerative disease. Stage 1: Physiological Homeostasis (Reversible Compensatory Stage). Under healthy physiological conditions, ramified microglia act as immune sentinels of the central nervous system (CNS), maintaining iron homeostasis in the microenvironment. They primarily take up transferrin-bound iron (Tf-Fe^3+^) via transferrin receptor (TfR) and endosomal divalent metal transporter 1 (DMT1). Intracellular iron metabolism is tightly regulated by the IRP/IRE pathway, driving high baseline levels of ferritin synthesis. The internalized iron is safely chelated within intact ferritin complexes, providing a crucial metabolic buffer; simultaneously, constitutively expressed membrane iron exporter ferroportin 1 (FPN1) maintains iron efflux balance, preventing accumulation of free iron. Stage 2: Acute Compensatory Phase (Injury or Early Stress). In response to acute microenvironmental changes, microglia activate protective compensatory mechanisms. By synergistically upregulating iron uptake proteins (increased DMT1 expression), ferritin synthesis, and efflux proteins (increased FPN1 expression), they expand the cell’s buffering capacity against pathological metal ions. During this phase, the intact iron buffering mechanism effectively blocks the Fenton reaction, protecting surrounding vulnerable neurons from acute oxidative damage. Stage 3: Chronic Toxicity Cascade and Decompensation (Malignant Cycle Phase). As metal buffering mechanisms collapse, microglia undergo profound metabolic and transcriptional reprogramming, shifting toward a high-inflammatory and ferroptosis-susceptible phenotype.

Therefore, this “metal-inflammation axis” serves as a fundamental pathological bridge in the brain: after acute sterile injury, the collapse of the metal buffering mechanism in microglia will continuously “prime” the neuroinflammatory microenvironment. This lingering neuroinflammation then acts as an upstream catalyst, sowing and driving the protein aggregation and chronic neurodegenerative cascades that will be discussed later (Figure 3).

Iron Traps and LIP Formation: Persistent pro-inflammatory factor feedback inhibits FPN1 expression, cutting off iron export pathways (“iron trap”). Extreme intracellular iron overload causes ferritin to become completely saturated, triggering lysosome-mediated ferritinophagy and lysosomal membrane permeabilization (LMP), releasing large amounts of free ferrous iron (Fe^2+^) and forming a highly destructive labile iron pool (LIP).

Ferroptotic Stress: Mitochondrial dysfunction and upregulated glycolysis generate abundant endogenous H_2_O_2_, which, combined with LIP, quickly catalyzes the Fenton reaction, sharply amplifying the ROS signal. Simultaneously, glutathione (GSH) depletion, GPX4 inactivation, and the accumulation of polyunsaturated fatty acids (PUFAs) mediated by ACSL4 collectively trigger lethal lipid peroxidation, leading to ferroptotic stress in microglia.

Non-Cell-Autonomous Neurotoxicity: Microglia continuously release pro-inflammatory factors (TNF-α, IL-1β), forming a “inflammation-iron accumulation” malignant feedback loop through the NF-κB pathway. Toxic ROS and Fe^2+^ that diffuse outward not only directly damage synaptic structures and upregulate neuronal iron transport receptors to induce non-cell-autonomous neuronal death but also drive the oligomerization and aggregation of pathological proteins such as Aβ, Tau, and α-synuclein through direct oxidative modification and autophagy inhibition. In addition, damaged neurons and synaptic debris further produce reverse toxicity, exacerbating lysosomal damage in microglia and amplifying microenvironmental inflammation, ultimately promoting the continuous progression of chronic neurodegenerative pathology.

## 4 The effects of metal ions on neurodegenerative diseases

### 4.1 Convergent pathological pathways of metal homeostasis imbalance

Although Fe, Cu, and Zn generally play a role in the onset and progression of CNS diseases by inducing oxidative stress, their intracellular toxic kinetics exhibit a high degree of spatial and mechanistic heterogeneity. Iron overload triggers the classic ferroptosis cascade mainly at the ER by interfering with NCOA4-mediated ferritin autophagy and causing GPX4 depletion (Gao et al., 2023). In stark contrast, pathological copper accumulation directly targets mitochondria by specifically binding to acylated matrix proteins in the tricarboxylic acid (TCA) cycle and triggering abnormal aggregation, thereby initiating copper-induced cell death—a cell death pathway that is completely independent of apoptosis (Tsvetkov et al., 2022). At the synapse level, abnormally released vesicular zinc ions accumulate heavily in the synaptic cleft, and by over-activating extrasynaptic NMDA receptors and mediating abnormal CDK5 phosphorylation, they directly drive excitotoxic damage to postsynaptic neurons (Krall et al., 2021).

**FIGURE 4:**
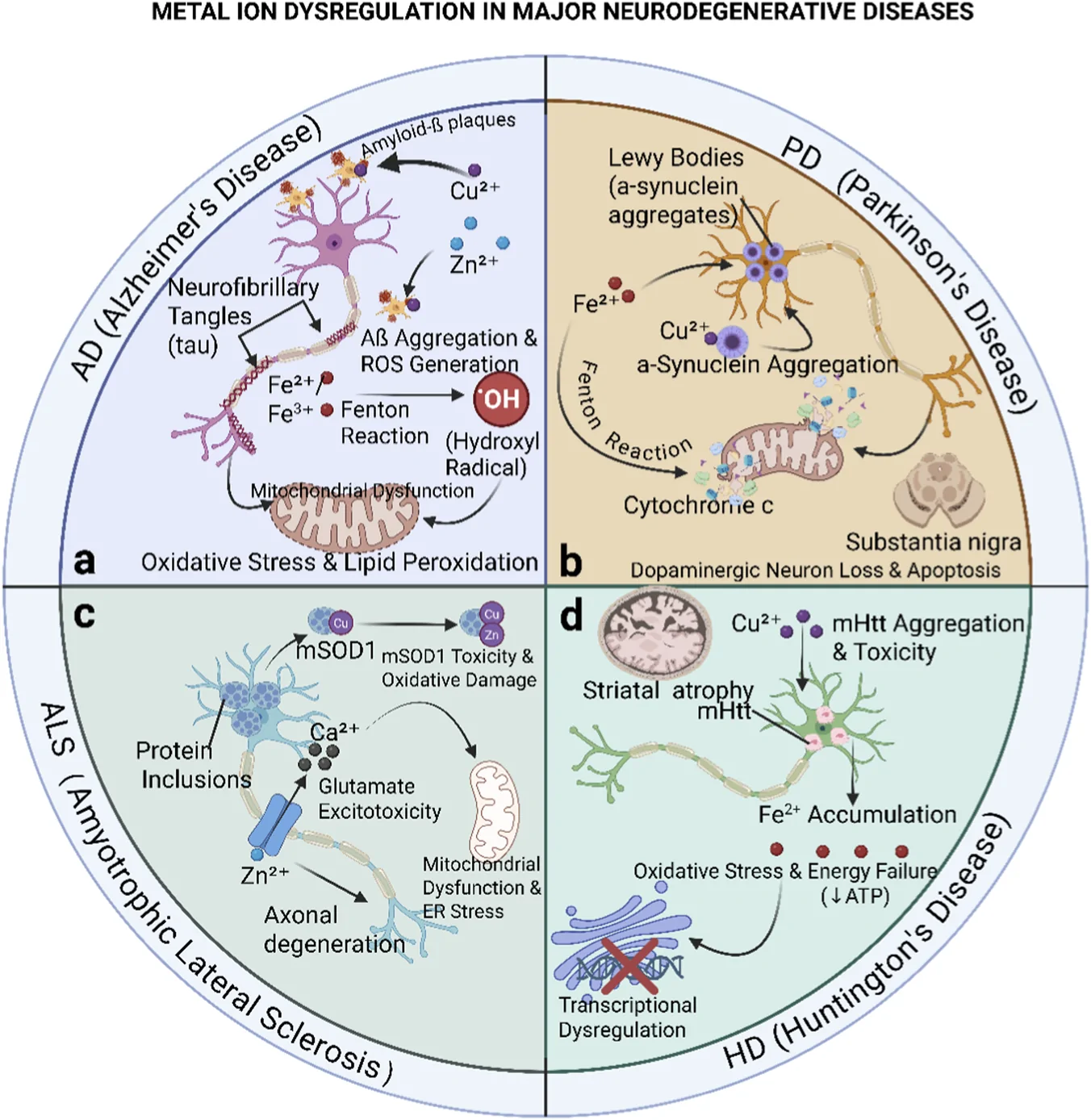
Metal ions and major neurodegenerative diseases. **(a)** Metal ions and AD. In AD, Cu^2+^, Zn^2+^, Fe^2+^/Fe^3+^ drive β-amyloid (Aβ) aggregation and tau protein hyperphosphorylation to form neurofibrillary tangles. The Fe^2^ mediated Fenton reaction produces·OH, triggering oxidative stress, lipid peroxidation and mitochondrial dysfunction. **(b)** Metal ions and PD. Fe^2+^ and Cu^2+^ promote the aggregation of α-synuclein into Lewy bodies; the Fe^2+^-driven Fenton reaction generates ROS, leading to the loss of dopaminergic neurons and apoptosis induced by the release of cytochrome c. **(c)** Metal ions and ALS. The dysregulation of Zn^2+^ and Cu^2+^ intensifies the toxicity of mutant SOD1. Ca^2+^ mediates the excitatory toxicity of glutamate, causing mitochondrial dysfunction, endoplasmic reticulum stress and axonal degeneration. **(d)** Metal ions (such as Cu^2+^ and Fe^2+^) promote the aggregation and toxicity of mutant Huntington protein (mHtt), while the accumulation of Fe^2+^ leads to oxidative stress and energy depletion (reduced ATP production), resulting in transcriptional dysregulation and striatal atrophy. Overall, metal ion dysregulation plays a significant role in neuronal damage and disease progression in neurodegenerative disorders by driving key pathways such as abnormal protein aggregation, oxidative stress, mitochondrial dysfunction, and excitotoxicity.

Although the aforementioned upstream initiating events possess unique subcellular targeting, their pathological effects ultimately converge inevitably onto a common downstream interconnected network. Initial oxidative damage induced by metal ion imbalance irreversibly activates microglia and astrocytes, leading to persistent chronic neuroinflammation. This highly inflammatory microenvironment creates a vicious cycle with mitochondrial damage, making the energy collapse in neuron clusters worse. More importantly, this long-term metal toxicity environment not only directly causes the loss of neurons, but also inhibits the endogenous neural stem cell pool, thereby damaging the repair and regeneration capabilities of the CNS. Therefore, systematically elucidating these convergent pathogenic cascades is a fundamental prerequisite for transcending traditional classifications and developing universal metal-targeted intervention strategies (Figure 4).

### 4.2 Impact of metal ions on AD

Iron was the first metal reported to be associated with changes in AD. Abnormal iron deposition in the brains of AD patients can induce the massive production of ROS via the Fenton reaction, which in turn leads to oxidative stress and triggers neuronal cell death pathways (Gao et al., 2025). The extracellular accumulation of amyloid-β (Aβ) protein is a pathological hallmark of AD patients, and neurofibrillary tangles (NFT) are formed by the intracellular deposition of highly phosphorylated tau protein (Tiwari et al., 2019). Aβ and tau proteins have a synergistic effect in the pathogenesis of AD, causing motor, cognitive and perceptual disorders in patients (Zhang et al., 2021). The accumulation of Fe will aggravate the incorrect folding of Aβ and the excessive phosphorylation of tau, leading to AD. Moreover, the excessive phosphorylation of tau will cause a disorder in the iron excretion mechanism in the brain, resulting in the accumulation of Fe in the brain and further exacerbating brain damage, forming a vicious cycle (Rogers et al., 2008; Lei et al., 2012; Bandyopadhyay and Rogers, 2014). Astrocytes and microglia release inflammatory factors in response to iron accumulation, thereby accelerating the progression of AD (Liu S. et al., 2022; Wang F. et al., 2022).

Cu is involved in glial cell apoptosis, and abnormal synaptic regulation of it causes memory and learning dysfunction (Kalita et al., 2017). Cu can affect synaptic function. During the excitatory synaptic activity process, copper-containing vesicles undergo activity-dependent release from the presynaptic membrane. On the postsynaptic membrane, copper ions act as a non-competitive inhibitor to regulate the function of N-methyl-D-aspartate receptors (NMDAR) and the activity of glutamate-activated α-amino-3-hydroxy-5-methyl-4-isoxazolepropionic acid receptors (AMPAR), thereby significantly inhibiting glutamate-mediated neural transmission (Dodani et al., 2014). Research has found that copper (Cu) is involved in the occurrence and development of AD. In the brain tissues of AD patients, Cu homeostasis imbalance has been discovered, manifested as abnormal fluctuations in copper ion concentration levels and pathological changes in their spatial localization. Both amyloid precursor protein (APP) and Aβ peptide have copper binding sites, and their interaction with copper may lead to potential neurotoxicity by generating ROS. The Cu metabolism of AD patients is abnormal, which may also affect the disease process of AD (Mathys and White, 2017). The research has found that the harmful effects of copper mainly lie in its uncontrolled binding with proteins inside cells, which promotes the misfolding and aggregation of proteins, leading to necrosis. GSH has antioxidant properties. The study has discovered that Cu can accelerate the depletion of GSH through the rapid influx of Ctr1, which allows Cu to freely combine with intracellular proteins and cause toxicity, leading to oxidative stress (Ho et al., 2022). The regulation of steady-state copper levels depends on the liver; studies have shown that copper regulation relies on copper-containing enzymes that use copper as a cofactor, as well as copper transport proteins that transport the metal (Sensi et al., 2018), ATP7B is a transport ATPase that mediates the assembly of Cu to new Cp in hepatocytes. Its activity is crucial for maintaining copper homeostasis. Free copper (nCp-Cu) that fails to bind to ceruloplasmin has a high redox activity and is prone to induce cytotoxicity by catalyzing the generation of free radicals (Sensi et al., 2018). In AD, the impairment of ATP7B can affect the overall levels of nCp-Cu throughout the body, which in turn drives or exacerbates the oxidative stress pathological process of the disease (Squitti et al., 2016). Copper overload can lead to mitochondrial copper accumulation, and alterations in mitochondrial copper levels are implicated in the development of neurodegenerative diseases and signal transduction (Swerdlow, 2009). The abnormal accumulation of copper ions in lysosomes can drive the progression of AD. Excessive copper can induce lysosomal dysfunction (such as causing membrane permeabilization or inhibiting the activity of hydrolases), thereby leading to the inhibition of the autophagy-lysosome pathway (ALP), ultimately resulting in the ineffective clearance of neurotoxic Aβ and Tau protein aggregates and their pathological deposition (Orr and Oddo, 2013; Polishchuk and Polishchuk, 2016).

Copper, as an indispensable catalytic cofactor, participates in redox and various metabolic activities in the brain. It endows multiple key target enzymes, including ceruloplasmin (Cp), SOD1, and cytochrome c oxidase (CcO), with structural stability and catalytic activity (Gale and Aizenman, 2024). The Ctr1 regulates the transport of copper in all cells. The copper transport ATPase A (ATP7A)/copper transport ATPase B (ATP7A/B) regulates the excretion of copper in cells. Studies have found that the ATP7B (K832R) mutation can lead to copper accumulation or deficiency in cells, increasing the risk of AD and causing the loss of ATP7B function (Mercer et al., 2017). In AD patients, the unstable copper pools (LCPs) in the cytoplasm expand, serving as the upstream trigger for the pathological changes of tau protein; excessive active copper ions significantly upregulate the activities of the CDK5 and GSK3β kinase pathways, thereby mediating and accelerating the cascade of tau protein hyperphosphorylation (Kitazawa et al., 2009; Lei et al., 2021; Chakraborty et al., 2024). Aβ plaques represent a core pathological hallmark of AD. Research has revealed significantly elevated Cu content within Aβ plaques in AD patients, while Cu levels in tissues surrounding Aβ plaques are lower than normal, whereas no difference in cerebrospinal fluid (CSF) copper levels is observed between AD patients and normal controls (Adlard et al., 2008; Wang et al., 2015; Mathys and White, 2017). Transcription factor Sp1 acts as a key effector molecule responsive to changes in the intracellular copper microenvironment. Copper overload aberrantly activates Sp1 transcriptional activity, leading not only to direct upregulation of β-site amyloid precursor protein cleaving enzyme 1 (BACE1) expression to drive Aβ generation but also to binding and activation of the microtubule-associated protein tau (MAPT) gene promoter. Thus, Cu homeostasis disruption simultaneously exacerbates Aβ accumulation and pathological Tau enrichment at the gene expression level via the common Cu-Sp1-BACE1/MAPT transcriptional regulatory axis, exerting upstream driving effects on the dual core pathologies of AD (Hill et al., 2023; Zheng et al., 2025).

Both Wilson disease (WD) and a subset of AD patients exhibit pathological elevation of systemic nCp-Cu. Zinc induces metallothionein and inhibits intestinal copper absorption, thereby restoring free copper homeostasis. Based on this mechanism, zinc therapy is used for WD. Given the similarity between WD and AD, employing zinc therapy as an intervention to correct nCp-Cu imbalance may offer a promising translational direction for AD clinical treatment (Squitti et al., 2020). AD is characterized by Aβ deposition in the brain parenchyma, including the neocortex and hippocampus, as well as in cerebral blood vessels. APP, a type I transmembrane glycoprotein possessing a copper/zinc-binding domain (belonging to the metalloprotein family), is associated with Zn homeostasis disruption and abnormal intracellular zinc mobilization in AD pathology (Huang et al., 2000). Prominent extracellular deposits in AD brains consist primarily of Aβ and biometal ions (zinc, copper, iron). Zinc ions affect the formation of amyloid plaques, suggesting that maintaining Zn homeostasis in the CNS may represent a potential therapeutic target for AD (Kozin, 2023). Microglia, as immune cells in the CNS, have been shown to phagocytose Aβ and Tau proteins, thereby slowing AD progression. Additionally, microglia can form physical barriers around Aβ, attenuating Aβ neurotoxicity (Frackowiak et al., 1992; Qiu et al., 1997; Condello et al., 2015; Yuan et al., 2016). However, microglia may also exacerbate AD progression, as activated microglia exhibit pro-inflammatory functions, and the Aβ and Tau proteins they phagocytose may be transferred to uninfected neurons, thereby worsening AD pathology (Sosna et al., 2018; Brelstaff et al., 2021; d’Errico et al., 2022; Thakur et al., 2023). Activation of the NLRP3 inflammasome is a major factor in neuroinflammation (Dempsey et al., 2017). Studies have shown that zinc enters microglial cells via the zinc transporter ZIP1, triggering hemichannel-mediated ATP release that activates P2X7 purinergic receptors (P2X7) in an autocrine/paracrine manner. Activated P2X7 receptors induce microglial activation through the nicotinamide adenine dinucleotide phosphate (NADPH) oxidase/poly (ADP-ribose) polymerase 1 (PARP-1) signaling axis. Moreover, zinc can directly activate the NF-κB signaling pathway in microglia, a core transcriptional driver of neuroinflammation, resulting in a phenotype similar to LPS-induced microglial activation (Kauppinen et al., 2008; Higashi et al., 2011). Activated microglia promote the release of inflammatory factors such as TNF-α and IL-6, intensifying the inflammatory microenvironment within the brain, directly damaging neurons and promoting the pathological toxicity of Aβ and tau (Shippy et al., 2024). Therefore, current research shows that zinc has a bidirectional function in regulating microglia.

### 4.3 Impact of metal ions on PD

The main pathological manifestations of PD are the accumulation of α-synuclein and the loss of dopaminergic neurons in the substantia nigra pars compacta (SNpc) (Tysnes and Storstein, 2017). Studies have shown that both oligomers and aggregates of α-Syn are toxic. The binding of Fe to α-Syn significantly promotes its oligomerization and subsequent aggregation, leading to selective alterations in synaptic proteins and ultimately impairing the electrophysiological excitability of neurons and the connectivity of synaptic networks (Volpicelli-Daley et al., 2011; Cheng et al., 2022). Microglia are resident immune cells in the brain, possessing functions such as phagocytosis, immune monitoring, and immune protection. They are associated with various neurodegenerative diseases. Fe is related to the activation of microglia. Accumulation of iron in the cells causes microglia to transform from the M2 phenotype to the M1 phenotype, accelerating the progression of PD (Yu et al., 2023). Excessive iron in cells can not only generate ROS through the Fenton reaction, but also activate arachidonic acid 15-lipoxylase (ALOX15), promoting the oxidation of polyunsaturated fatty acids. Through animal experiments, it has been found that PD models induced by MPTP (PD animal model modeling agent) can improve symptoms by using iron chelating agents (such as deferoxamine (DFO)). It is suggested that Fe has a potential therapeutic effect in the PD pathological model (Liu et al., 2025). The abnormal expansion of the intracellular LIP is more common in the brains of PD patients, causing neuronal ferroptosis and degenerative changes. Researchers have blocked the outbreak of ROS and saved neuronal ferroptosis by targeting and removing iron from LIP (through chelation), and this represents a potential new therapeutic target for PD (Lei et al., 2024).

Copper mainly triggers or induces PD through oxidative stress, dopamine oxidation, and promoting the aggregation of α-synuclein. Moreover, studies have found that copper deficiency and the formation of SOD1 aggregates are associated with the progression of PD (Bisaglia and Bubacco, 2020). Studies have shown that copper deficiency in the substantia nigra and locus coeruleus can lead to the de-metallation of SOD1, resulting in the formation of unstable apo-SOD1 proteins; these proteins are prone to form amorphous aggregates, thereby losing their antioxidant neuroprotective function (Trist et al., 2017). COMMD3 belongs to the copper-metabolizing protein family and is an important component of the CCC subunit (containing CCDC22/CCDC93) in the Commander complex, responsible for regulating the activity of lysosomal glucocalosidase (GCase) (Minakaki et al., 2025). Since the GCase functional defect is a key risk factor for PD, this suggests that the COMMD protein family may serve as a hub connecting copper homeostasis and lysosomal function. Reduced copper and increased iron levels have been observed in the substantia nigra and caudate nucleus of patients with PD. Since copper is a key cofactor in regulating the activity of iron-containing enzymes (such as ceruloplasmin), reduced bioavailability of copper in the brain can lead to impaired iron metabolism, resulting in abnormal iron accumulation and associated oxidative stress (Montes et al., 2014).

Zinc ions (Zn^2+^) influence the pathological progression of vascular dementia (VD) and PD (Mizuno et al., 2024). Zn is involved in the occurrence and development of PD (Nishio et al., 2022), it has been established that dopaminergic neuron loss and microglial activation are closely associated with PD pathogenesis (Isik et al., 2023). Zinc homeostasis is essential for maintaining neuronal function. Research has shown that hyperactivation of the cortico-striatal glutamatergic system is a key feature driving dopaminergic neurotoxicity in PD, and vesicular zinc in the forebrain, acting as a neuromodulator in cortical neurons, cooperates with glutamate. By eliminating synaptic vesicular zinc in the mouse nervous system, investigators found that PD symptoms were significantly alleviated, suggesting that PD may be associated with Zn accumulation at synapses (Sikora and Ouagazzal, 2021). Levodopa is a commonly used clinical treatment for PD; studies have found that frequent levodopa use leads to decreased serum zinc levels, suggesting that preventing serum zinc deficiency may be important in PD management (Matsuyama et al., 2021). Astrocytes produce antioxidant molecules metallothioneins (MT)-1/2 under oxidative stress; these cysteine-rich proteins protect neurons from oxidative damage. MTs bind metals such as Zn and Cu, and also mediate transcriptional activation of various genes by regulating free zinc levels. Therefore, targeting zinc-binding proteins in astrocytes may represent a novel therapeutic strategy for PD (Miyazaki and Asanuma, 2021). The research has found that zinc oxide nanoparticles (ZnO-NPs) can intervene in various fundamental mechanisms related to the onset of PD, mainly by increasing the expression of neurotrophic factors, inhibiting the formation of amyloid fibers and tau phosphorylation, reducing oxidative stress, and inhibiting cell apoptosis and neuroinflammation (Tang K. S. et al., 2024). These effects all suggest that zinc holds significant potential for the treatment of PD.

### 4.4 Impact of metal ions on ALS

The core pathology of ALS is the selective degeneration of motor neurons in the brain and spinal cord. Ferroptosis mediates the key pathway for the specific death of motor neurons in ALS (Wang et al., 2022b). In ALS, it was found that the key antioxidant enzyme GPX4, which inhibits ferroptosis, was significantly downregulated. SPY1 is a “cyclin-like” protein that can reverse the imbalance of the GCH1/BH4 axis (the resistance axis of ferroptosis) and inhibit lipid peroxidation induced by transferrin receptor protein 1 (TFR1), thereby inhibiting neuronal ferroptosis in ALS and restoring the activity of GPX4 (Wang D. et al., 2023). In general, iron levels in ALS patients and mouse models are significantly elevated. This pathological environment is closely related to the abnormal accumulation of extracellular glutamate-high concentrations of glutamate competitively inhibit the activity of the cystine glutamate reverse transporter (System Xc-), hinder cystine uptake and deplete GSH, ultimately inducing oxidative stress and neuronal iron death (Wang et al., 2024). In other tissues, iron is released from ferritin through ferroptosis and then made available for cellular use. However, with aging, the degradation of iron autophagy in skeletal muscle leads to some muscle disorders. As the disease progresses, the iron content in the muscle tissues of ALS patients and their animal models continues to increase, which may be related to the imbalance in iron metabolism (Halon-Golabek et al., 2018; Ru et al., 2025). A close association has been observed between iron levels and microglial activation in ALS patients, suggesting that modulating iron levels may improve microglial function and thereby alleviate clinical symptoms of ALS (Gao et al., 2024; Liddell et al., 2024). Therefore, iron is widely involved in the pathological cascade reaction in the pathogenesis and progression of ALS, whether in the CNS, muscle tissue or microglia.

Cu is a core cofactor that maintains the catalytic activity of SOD1. It has antioxidant and anti-oxidative stress effects in the body. In patients with familial amyotrophic lateral sclerosis (fALS), mutations in the SOD1 gene have occurred, leading to the clinical symptoms of ALS. This suggests that copper affects the progression of fALS (Valentine et al., 2005). The research has found that protein folding errors are the main cause of ALS in patients with SOD1 mutations and in the mouse models. During the folding process of SOD1, there is a crucial step - the copper partner (CCS) of SOD1 can insert into the catalytic copper cofactor and oxidize the disulfide bonds within its subunits to modify the newly formed SOD1 polypeptide. The obstruction of SOD1 maturation leads to an increase in the content of immature SOD1, thereby inducing or exacerbating ALS. The degree of copper deficiency in SOD1 aggregates is considered to be proportional to the clinical severity of ALS (Seetharaman et al., 2009; Trist et al., 2022). Copper homeostasis significantly influences ALS onset and progression. Studies have shown a close association between copper metabolism disruption in skeletal muscle and ALS in mouse models. By training ALS mice to exercise, researchers improved skeletal muscle metabolism, thereby altering copper metabolism, and found that ameliorating copper metabolism had positive effects on ALS (Sauzéat et al., 2018; Białobrodzka et al., 2024). Disruption of cellular copper homeostasis and copper enzyme activity may exacerbate SOD1 mutant protein toxicity and disease progression. Use of the copper chelators tetrathiomolybdate (TTM) and D-penicillamine has been shown to significantly reduce neuronal death in animal models, exerting beneficial effects in ALS animal models (Zhu et al., 2024).

ALS is associated with certain trace metals. Studies have found that Se and Zn levels in the body are inversely correlated with ALS, suggesting that dysregulation of metal homeostasis, including Zn, is a critical factor driving ALS progression (Peters et al., 2016). Many cases of fALS are associated with SOD1 mutations. Since SOD1 is a typical copper- and zinc-dependent metalloenzyme, this has led researchers to suspect that ALS is linked to metal ions. Concurrently, studies have found that insoluble SOD1-enriched fractions are not enriched with copper and zinc, indicating a potential therapeutic value in restoring Cu/Zn homeostasis in fALS (Lelie et al., 2011). ROS can induce oxidative stress, leading to fibril-like aggregation and misfolding of the SOD1 protein. Such aberrant SOD1 protein is involved in disease progression in both sporadic ALS (sALS) and fALS. Hydrogen peroxide (H_2_O_2_), a product of SOD1-catalyzed reactions, can act as a substrate at pathological concentrations to trigger the misfolding trajectory and toxicity of SOD1 in ALS pathogenesis (Sanghai and Tranmer, 2021). Long-term exposure to metal environments (such as Zn, Cu, etc.) leads to a significant increase in the metal content in the blood and urine, and is associated with an increased risk of ALS and a decreased survival rate. This suggests that abnormal metabolism of metal ions is involved in the occurrence and prognosis of ALS (Jang et al., 2025).

### 4.5 Impact of metal ions on HD

Huntington’s disease (HD) is an autosomal dominant disease caused by repeated amplification (≥36) of cytosine adenine-guanine (CAG) in the Huntington (HTT) gene. The main manifestation is the development of chorea and dysfunction of muscle tone, and ultimately the development of bradykinesia. Current studies have shown that the pathological process of HD is closely related to the imbalance of iron regulation, and obvious iron accumulation has been found in neurodegenerative areas of HD (Macdonald, 1993; Muller and Leavitt, 2014). Mutated HTT leads to increased iron influx into cells, causing an imbalance in intracellular iron homeostasis and resulting in oxidative stress (Muller and Leavitt, 2014). In patients with HD, the phenomenon of myelin breakdown occurs, which may be caused by the increase of iron levels in oligodendrocytes, thereby further leading to myelin breakdown and neurotoxicity, forming a vicious cycle (Casella et al., 2020). Both *in vitro* and *in vivo* studies have demonstrated significant iron accumulation in the brains of HD patients, suggesting a close association between HD and Fe (Van Den Bogaard et al., 2013). Mitochondria, as the main site for ATP production, exhibit abnormal mitochondrial iron metabolism in mouse HD models and human HD patients. The use of the iron chelator DFO can counteract the HD symptoms caused by abnormal mitochondrial iron metabolism. Therefore, targeting iron clearance may be a potential approach for treating HD (Agrawal et al., 2018).

Surveys indicate that HTT mutations are present in HD patients many years prior to disease onset. Even at stages when no typical motor or cognitive symptoms are manifested (i.e., the premanifest stage), structural changes are already detectable in the brains of mutation carriers (Johnson et al., 2021). Studies have found elevated iron levels, manifested as increased R1 and R2* values, in the globus pallidus (GP) and putamen of premanifest HD (preHD) gene carriers (Van Den Bogaard et al., 2013; Johnson et al., 2021). Furthermore, compared with healthy controls, significantly higher iron deposition rates have been observed in premanifest HD patients closer to disease onset and in patients with early-stage HD. Therefore, monitoring brain iron content may provide further insights into the pathophysiological changes in HD and serve as a measurable indicator for clinical trials (Chen et al., 2019).

Elevated levels of copper and iron are present in the brains of both HD patients and mice expressing the HD gene (Fox et al., 2007). Significant neuronal loss has been observed in regions such as the striatum and cortex of HD patients, and this neuronal loss is associated with mutant huntingtin protein (mHTT). The abnormal expansion of the polyglutamine repeat within mHTT leads to the formation of neurotoxic aggregates within cells. It has been postulated that copper accumulation in the brains of HD patients promotes mHTT aggregation and disease progression; conversely, targeted depletion of environmental copper effectively reverses the formation of mHTT oligomers and aggregates (Wang et al., 2025). Autophagy adaptor proteins help maintain nervous system homeostasis by recognizing and clearing toxic mHTT aggregates. Clinical and imaging studies have confirmed significant copper homeostasis dysregulation in the brains and body fluids of HD patients (Rosas et al., 2012; Pfalzer et al., 2022). Further research has shown that abnormal copper accumulation interferes with autophagic clearance pathways and significantly enhances mHTT aggregation toxicity in *Drosophila* models of HD by preferentially promoting the formation of more toxic small aggregates and increasing the accumulation of Thioflavin S-positive β-sheet (ThS^+^ β-sheet) amyloid structures (Lobato et al., 2024). Microglia are essential for maintaining nervous system function; however, excessive microglial activation can trigger neuroinflammation, leading to neurological abnormalities. Studies have indicated that abnormal heavy metal levels in the brain can activate microglia and induce neuroinflammation (Tizabi et al., 2024).

### 4.6 Impact of metal ions on other neurodegenerative diseases

Friedreich’s ataxia (FA) is caused by mutations in the FXN gene on the chromosome. The FXN gene is involved in the formation, maintenance, and repair of Fe-S clusters and in the regulation of oxidative stress in the brain (Koeppen, 2011; Gao et al., 2025). Studies have found that under iron homeostasis, healthy cells promote the production of Fe-S clusters and inhibit the import of additional iron through iron regulatory proteins (IRP1 and IRP2). However, in FA, even if the iron status is normal, the formation of Fe-S clusters is weakened, and other iron regulatory systems (IRP1, TfR, and ferritin) are also dysregulated, resulting in an imbalance in iron homeostasis and oxidative stress (Cheng et al., 2022). Pantothenate kinase-associated neurodegeneration (PKAN) is a hereditary progressive disorder characterized by brain iron accumulation and glial cell degeneration. Although clinical studies have shown that the iron chelator DFO can effectively reduce MRI signals of iron deposition in the brain, there remain significant challenges in achieving substantial improvement in core neurological symptoms (Hartig et al., 2012). β-propeller protein-related neurodegenerative disease (BPAN) is a brain iron accumulation neurodegeneration caused by the deletion of WDR45. Therefore, regulating brain iron metabolism may be one of the means to improve the symptoms of patients (Diaw et al., 2022).

In addition to the classic neurodegenerative paradigm driven by Fe, Cu, and Zn, the pathological trajectories of manganese, Se and Ca also provide unique mechanistic insights into the chronic deterioration of neural functions. The pathological accumulation of Mn exhibits a high degree of structural selectivity; it preferentially targets the basal ganglia, inducing selective mitochondrial dysfunction and oxidative stress within dopaminergic neurons, thereby establishing a direct mechanistic link to manganism—a disease entity that is clinically distinct from Parkinson’s disease but highly overlapping with it in terms of pathophysiology. At the same time, Se acts as a key upstream regulator of protein toxicity; Long-term deficiency of trace elements will weaken the enzymatic activity of endogenous selenium proteins, thereby failing to effectively neutralize lipophilic peroxides. This, in turn, actively accelerates the abnormal hyperphosphorylation and fibrillar aggregation of tau protein and α-synuclein protein. This protein toxicity cascade works together with a catastrophic Ca^2+^ balance disruption. Chronic neurodegenerative stress leads to sustained, excessive activation of voltage-gated calcium channels and NMDA receptors in neurons, driving an unchecked influx of extracellular Ca^2+^ into the cytoplasm. This persistent calcium overload structurally breaks down the mitochondrial cristae, compromises cellular bioenergetics, and permanently triggers the downstream deadly calpain-caspase pathway, directly leading to synapse cutting and irreversible neuron loss (Turovsky et al., 2024).

### 4.7 The role of metal homeostatic imbalance in neural regeneration and stem cell fate

In addition to causing neurotoxicity, metal homeostasis imbalances also actively impair the CNS’s endogenous regenerative capacity. This is a key aspect that’s often overlooked in pure neuroprotection strategies. Realizing true neural regeneration requires the coordinated proliferation of neural stem cells (NSCs) and the differentiation of oligodendrocyte precursor cells (OPCs) for myelin regeneration. Zinc is a double-edged sword in this context: physiological zinc is a prerequisite for brain-derived neurotrophic factor (BDNF) signaling and adult neurogenesis (Burnstock, 2013). And its pathological accumulation will weaken synaptic plasticity. Also, the process of myelin regeneration really depends on iron, because iron is an essential cofactor for the synthesis of myelin lipids (Stephenson et al., 2014). Therefore, non-selective metal chelation poses a significant risk to the enzymatic process required for the reconstruction of the “starvation” neural circuit. Therefore, a true regeneration strategy must prioritize the restoration of physiological metal distribution in space and time, rather than systemic depletion.

## 5 Recent advances in research on drugs related to metal elements

Given that the imbalance of metal ion homeostasis exists in the pathological evolution of many nervous system diseases, targeted correction of this microenvironment (such as metal chelation or element supplementation) has become a potential therapeutic approach. Currently studied metal targeting strategies mainly include metal ion chelators, zinc preparations and multi-target ligands. However, in practical applications, due to the contradiction between BBB permeability and target specificity and metal steady-state interference, different intervention strategies have significant differences in clinical transformation.

### 5.1 Iron chelating agents and manganese removal

The Fenton reaction driven by iron overload and cellular ferroptosis are common pathogenic mechanisms underlying traumatic brain injury (TBI), stroke (CVA), and neurodegenerative diseases. Consequently, research on iron chelators is most extensive in the field of neurological disorders.

The earliest iron chelator used in clinical practice, DFO, can effectively inhibit ferroptosis mediated by NCOA4. In ischemia-reperfusion injury and TBI animal models, DFO can inhibit oxidative stress and rescue metabolic disorders (Qiu et al., 2024; Zhao et al., 2024; Zhu et al., 2026). However, DFO has poor lipid solubility (LogP = −2.2) and an extremely short half-life (20–30 min), with limited penetration of the BBB. In response to this problem, the researchers developed a ROS-responsive DFO hydrogel system that increases drug concentrations in the brain through targeting and shows good effects in the TBI model (Qiu et al., 2024). Deferiprone is a drug of considerable interest in iron chelation research; it possesses good lipophilicity and BBB permeability and has been extensively studied in clinical trials for brain iron deposition disorders such as PKAN and other NBIA conditions (Hartig et al., 2012). It has also been shown to reduce iron overload, reduce lipid peroxidation and gliosis in hemorrhagic stroke and TBI (Daglas et al., 2023; Fang et al., 2024). In the PD research, deferiprone showed positive signals in the early small-scale trials, but failed in the clinical trials: In the FAIR-PARK-II trial involving 372 early-stage PD patients who had not received levodopa treatment, although deferiprone successfully significantly reduced the iron content in the substantia nigra region (target binding confirmed) for these patients, the total MDS-UPDRS score after 36 weeks was significantly worse compared to the placebo group (Devos et al., 2022). Although the trial successfully achieved its target (reducing iron in the substantia nigra), clinical scores paradoxically worsened in the FAIR-PARK-II trial, offering a profound pharmacological caution (Devos et al., 2022). This clinical failure has exposed the fundamental flaw of the first-generation chelating agents: the inability to distinguish between the neurotoxic ‘labile iron pool (LIP)’ and the physiological iron needed for essential Fe-S cluster biosynthesis. By indiscriminately depleting iron from entire regions of the brain, deferiprone may have induced a secondary iron deficiency in the striatum, damaged the mitochondrial respiratory chain complex within the originally healthy neural network. This highlights a key paradigm shift: the next-generation of interventions must move away from “broad-spectrum chelation” and toward “spatiotemporally controlled metal redistribution,” selectively neutralize the extrastriatal LIP without starving essential metalloproteins.

Deferasirox is an oral long-acting iron chelator that has shown good results in the experimental treatment of neurodegenerative diseases. Its derivative has an inhibitory effect on kallikrein associated with neurodegenerative diseases (Boumali et al., 2025). Unlike DFO, the CNS penetration of deferasirox is limited. Therefore, it is suggested that its neuroprotective effect may rely more on the indirect consequences of peripheral iron metabolism regulation rather than direct targeted chelation within the brain. Salvianolic acid A is a natural iron chelator that can preserve nerve cells by activating the Akt/Nrf2 pathway and inhibiting ferroptosis, among other methods (Shi et al., 2024). The multi-target nature of these natural products provides a theoretical basis for further structural optimization.

The failure of these drugs to cross the BBB is closely related to their inherent physicochemical limitations. As summarized in Table 1, DFO has a high molecular weight and an unfavorable distribution coefficient (560.68 Da, LogP of −2.2), which fundamentally hinders its transport across the BBB. Conversely, although deferasirox has a more favorable LogP value (3.52), its extremely high plasma protein binding rate (PPB >99%) severely limits the amount of free drug that can cross the BBB and exert pharmacological activity. These data show that just having structural availability is not enough; without precise timing and location, high system exposure and poor brain bioavailability will inevitably lead to insufficient target site concentration, which also explains the poor clinical efficacy seen in trials like FAIR-PARK-II.

**TABLE 1:** Metal ion chelators and their use in the treatment of neurological disorders.

Medications	Target metal	Indications/research background	Core mechanism	Research phase	References	Physicochemical properties (MW [Da]/LogP)	Pharmacokinetics (PPB [%]/t_1_/_2_)
DFO	Fe	TBI, Ischemic stroke	Inhibits NCOA4-mediated ferritin autophagy; reduces the free iron pool	Preclinical/Phase II	Qiu et al. (2024), Zhao et al. (2024), Zhu et al. (2026)	560.68/−2.2	<10%/20–30 min (IV)
Deferiprone	Fe	Systemic iron overload (approved); hemorrhagic stroke,PD, PKAN (clinical trials)	BBB permeability; reduced lipid peroxidation; FAIR-PARK-II (PD, Phase II) showed target binding but symptoms worsened	Approved (peripheral iron overload, such as thalassemia)/Stage II–III (CNS disorders)	Hartig et al. (2012), Devos et al. (2022), Daglas et al. (2023), Fang et al. (2024)	139.15/0.17	<10%/1.9–3 h
Deferasirox (DFX) derivatives	Fe	Neurodegenerative diseases	Chelates free iron; stabilizes HIF-1α (during infection)	Preclinical	Boumali et al. (2025)	373.36/3.52	>99%/8–16 h
Salvianolic acid A (natural iron chelator)	Fe	Hemorrhagic stroke	Activate the Akt/Nrf2 pathway; inhibit ferroptosis	Preclinical	Shi et al. (2024)	494.41/1.9 (predicted)	>90%/1–2 h
D-penicillamine	Cu	WD (approved); ALS (experimental)	Promotes urinary copper excretion; counteracts the toxicity of SOD1 mutants	Approved (WD)/preclinical (ALS)	Zhu et al. (2024)	149.21/−1.7	∼80%/1.7–7 h
Ammonium tetrathiomolybdate (TTM)	Cu	ALS	Forms insoluble Cu ternary complexes; alleviates copper overload	Preclinical	Zhu et al. (2024)	260.28/N/A (inorganic complex)	Extremely high (forms ternary complex)/complex multi-compartment
PBT2 (8-hydroxyquinoline derivative)	Cu/Zn	AD (Phase II), HD (Phase II)	Redistributes metal ions within plaques; promotes the dissolution of Aβ/mHTT aggregates; crosses the BBB	Phase II	Lannfelt et al. (2008), Cherny et al. (2012), Huntington Study Group Reach2HD Investigators (2015)	350.5/2.8	>95%/12–16 h
Zinc acetate	Cu (indirect)	WD (approved); AD (exploratory)	Induces intestinal MT; competitively inhibits Cu absorption; corrects elevated nCp-Cu levels	Approved (WD)/exploratory (AD)	Squitti et al. (2020)	183.48/N/A (ionic salt)	∼60%/24–48 h (body pool)
CaNa_2_EDTA	Mn	Manganese poisoning/manganese accumulation in the nervous system	BBB has extremely poor permeability; it relies on the peripheral sink effect to passively promote the efflux of free Mn from the brain	Clinical applications	Vogt et al. (2025)	374.27/−3.86	<5%/20–60 min
Multi-target natural ligands (quercetin, curcumin, etc.)	Fe/Cu/Zn	Neurodegenerative diseases	Synergistic antioxidant effects of multi-metal chelation	Preclinical	Mazur et al. (2024), Chen et al. (2025)	302.23/1.5	>98%/3–10 h
Sodium selenite	Se	Ischemic stroke, AD	Activate the TFAP2c/Sp1 transcription program; specifically upregulate GPX4 expression to block lipophilic lipid peroxidation	Preclinical/Phase II	Alim et al. (2019)	172.94/N/A	N/A/12–24 h
Nimodipine	Ca	Traumatic brain injury, Ischemic stroke	Highly selective blockade of L-type voltage-gated calcium channels; suppression of cytoplasmic Ca^2+^ overload and secondary excitotoxicity	Approved (CNS vascular disorders)	Turovsky et al. (2024)	418.42/3.41	>95%/1.5–2 h

Molecular weight (MW), partition coefficient (LogP), plasma protein binding (PPB), and half-life (t_1/2_) values were sourced and aggregated from the DrugBank Database (Wishart et al., 2018) and PubChem (Kim et al., 2023).

As the current conventional manganese chelating agent, calcium disodium ethylenediamine tetraacetate (CaNa_2_EDTA) has some pharmacokinetic shortcomings: its BBB penetration ability is weak, consistent with its highly hydrophilic nature (LogP = −3.86), and manganese can only be eliminated in the peripheral circulation (Vogt et al., 2025). Its alleviation of manganese overload in the nervous system merely relies on the concentration gradient resulting from the reduction of manganese concentration in peripheral blood. It slowly and passively prompts the free manganese in the brain to flow outwards, but it is difficult to fundamentally reverse the established manganese accumulation in the brain tissue. Therefore, the development of novel chelating molecules with high CNS selectivity that can directly cross the BBB and facilitate *in situ* manganese clearance in the brain has become an urgent priority for the next breakthrough in this field.

### 5.2 Copper chelators and homeostasis modulators

Significant abnormalities in copper metabolism have been identified in neurodegenerative diseases, drawing insights from copper-targeted therapies for WD. D-Penicillamine and triethylenetetramine (TETA) promote urinary copper excretion and are first-line copper chelation therapies for WD. Ammonium tetrathiomolybdate (TTM) forms insoluble ternary complexes with copper, thereby reducing free copper levels, and has demonstrated neuroprotective effects in pathological models of ALS (Zhu et al., 2024). In ALS research, copper-targeting strategies using D-penicillamine or TTM have been shown to reduce mutant SOD1 toxicity and attenuate neuronal death induced by copper overload, providing a rationale for copper-targeted therapy in familial ALS (fALS) (Zhu et al., 2024).

PBT2 is a second-generation 8-hydroxyquinoline-based metal protein-attenuating compound (MPAC) with excellent BBB permeability and dual-targeting activity against Cu/Zn. Its mechanism of action involves redistributing mislocalized metal ions around Aβ plaques rather than simply chelating metal ions. In a phase II clinical trial for AD (Lannfelt et al., 2008), PBT2 significantly reduced cerebrospinal fluid (CSF) Aβ42 levels within 12 weeks—considered to reflect effective reduction of the total pool of soluble toxic Aβ oligomers in the brain—and improved performance on certain cognitive measures, including executive function, making it one of the most robust clinical translation examples of metal-targeted AD therapy to date (Lannfelt et al., 2008). This drug demonstrated good tolerability in the Phase II clinical trial of HD (the Reach2HD study), and some cognitive indicators (such as executive function) showed an improving trend. At the mechanism level, it promotes the disintegration of mutant Huntington protein mHTT aggregates by regulating and depleting abnormally accumulated metal ions such as copper (Cherny et al., 2012; Huntington Study Group Reach2HD Investigators, 2015).

Zinc acetate is an oral zinc preparation approved by the FDA for maintenance treatment of WD. It corrects the systemic increase in free copper (nCp-Cu) by inducing intestinal MT to competitively block copper absorption (Squitti et al., 2020). The main text has pointed out the similarities in nCp-Cu pathology between WD and AD. Based on this, the application value of zinc therapy in AD deserves further verification. The transformation logic where both AD and WD target the increase in nCp-Cu has strong mechanistic support.

### 5.3 Multi-target ligands and novel delivery strategies

The occurrence and development of neurological diseases are related to various metal ions. Therefore, the search for drugs with multi-metal targeting capabilities holds potential value. Clioquinol and PBT2 fall into this category; some active components of natural products have also been found to have synergistic neuroprotective effects through multi-metal chelation and antioxidation, such as quercetin, curcumin, and coumarin derivatives (Mazur et al., 2024; Chen et al., 2025). However, the permeability of BBB and the results of clinical trials still need to be optimized, systemic off-target toxicity is also a challenge that urgently needs to be addressed.

Due to the physical barrier posed by the BBB, nose-to-brain delivery has emerged as a cutting-edge drug delivery strategy. This route enables nanocarriers to bypass systemic clearance and deliver drugs directly to the CNS via the olfactory and trigeminal nerve endings (Lochhead and Thorne, 2012). In addition, by strategically introducing ROS-responsive chemical bonds into the polymeric backbone of the nanocarriers, not only does it ensure the structural stability of the drug-loading system during the normal physiological transport process, but it can also undergo specific cleavage in the highly oxidative stress environment of the ischemic penumbra microenvironment (Saravanakumar et al., 2017). This microenvironment-triggered degradation mechanism enables the localized, on-demand burst release of metal chelators in the lesion area. Thus, while maximizing the local bioavailability, it also minimizes the systemic off-target toxicity to the greatest extent.

To overcome the challenge of poor BBB penetration, researchers have developed various delivery strategies, with the ROS-responsive DFO-loaded hydrogel representing one such approach (Qiu et al., 2024). By utilizing responsive chemical bonds, these smart nanocarriers can rapidly disassemble in the oxidized microenvironment of the lesion site and deliver drugs on demand. In addition to penetrating BBB at the macroscopic level, achieving precise, subcellular-level targeted interventions is equally important, because metal toxicity mainly damages mitochondria, and the core cascade of lipid peroxidation and ferroptosis primarily originates within these organelles. Functionalizing the nanocarriers with specific mitochondrial-targeting peptides (such as the Szeto-Schiller (SS)-31 peptide) can provide them with significant advantages in pharmacokinetics. The SS-31 peptide can highly selectively target and bind to cardiolipin located on the inner mitochondrial membrane (IMM) (Szeto, 2014). This precise subcellular targeting allows the nano-delivery system to stabilize mitochondrial cristae under ischemic stress and directly neutralize excess ROS generated locally. This strategy effectively blocks the lethal Fenton reaction cascade, rescuing neurons from iron-dependent cell death pathways before irreversible cellular damage occurs. By developing novel targeted formulations to enhance the ability of chelating agents to cross the BBB, or to bypass the BBB entirely (For instance, by using the nasal administration route in combination with thermosensitive *in situ* hydrogels, achieving direct and rapid high-concentration accumulation in the CNS), this is also an important research direction in this field. A comprehensive summary of recent metal ion chelators and their targeted delivery strategies in neurological disorders is provided in Table 1.

### 5.4 The emerging metal-based nano-catalytic therapy (nanozyme)

To overcome the therapeutic limitations of traditional broad-spectrum chelating agents—particularly their poor penetration of the BBB, systemic toxicity, and disruption of physiological metal homeostasis—emerging metal-based nanocatalytic therapies have recently attracted significant attention. Unlike traditional chelators that passively isolate metal ions (usually at the risk of depleting essential metal proteins), metal nanoenzymes essentially mimic the catalytic activity of endogenous antioxidant enzymes (such as SOD and catalase), to continuously remove ROS without interfering with the endogenous trace metal pool.

Recent groundbreaking research has demonstrated the tremendous neuroprotective potential of these nanocatalytic platforms. For example, researchers designed inhalable phages loaded with cerium (Ce) nanozymes that can utilize M1 microglia as targeted cellular vehicles to specifically deliver and help relieve ischemic brain injury (Hong et al., 2025). Furthermore, by bypassing the BBB through non-invasive intranasal delivery, the mitochondria-targeted danphenolic acid B-Ce nanoenzyme has been shown to significantly enhance antioxidant defense and autophagy regulation in ischemic models (Li et al., 2026). In the context of acquired brain injury, nano-catalytic neuroprotection has also demonstrated remarkable efficacy in salvaging metabolic disorders and promoting the overall neurological function recovery after TBI (Zhu et al., 2025). These metal-based nanoenzyme strategies perfectly address the dual challenges of target specificity and maintaining physiological metal homeostasis, broadening the horizons for precise neuroprotective interventions.

### 5.5 Challenges and difficulties in clinical translation

Most treatments cannot cross the BBB. Currently clinically mature iron chelators such as DFO and deferasirox have problems such as high molecular weight and low lipid solubility, which leads to small amounts of drugs entering the lesion. Their neuroprotective effect relies to a certain extent on the indirect effect of peripheral iron metabolism. Although deferiprone possesses favorable BBB permeability, the negative results of the FAIR-PARK-II trial indicate that permeability alone is insufficient to predict clinical benefit. Therefore, the next-generation of CNS iron-targeted drugs must strike a more precise balance between permeability, selectivity, and safety.

Contradiction between target specificity and metal steady-state interference. Broad-spectrum chelators can not only clear overloaded metals, but may also interfere with the steady-state of other metals-for example, excessive chelation of iron by DFO may affect iron-sulfur cluster biosynthesis and mitochondrial respiratory chain function. Although PBT2 can double target Cu/Zn, it improves effectiveness but also increases the risk of interference with the steady-state of other metals. Developing next-generation ligands capable of selectively targeting pathological misfolded protein–metal complexes, rather than physiological metalloproteins, represents a central direction for improving the therapeutic window. Moreover, insights from the pathobiology study of the FAIR-PARK-II trial highlight that the appropriate disease stage for metal chelation is critical, rather than continuous chelation throughout the entire disease course. This underscores the necessity for future iron chelation clinical trials to strictly differentiate disease stages.

Given the extreme biochemical complexity involved in modulating the LIP using ROS-responsive nanocarriers and subcellular-targeting peptides (such as SS-31), predicting the off-target transcriptional toxicity of it in the body remains a formidable challenge. Specifically, iron overload drives classic ferroptosis by interfering with ferritin autophagy and depleting GPX4. Researchers can now perform virtual gene disruption (*in silico* knockout) modeling to precisely simulate how novel targeted nanomedicines dynamically reconfigure the gene regulatory networks of different glial cell and neuronal subpopulations (Kamimoto et al., 2023). This “digital twin” prediction paradigm, as an indispensable preclinical filter, establishes a safety boundary for toxic effects at the transcriptomic level before participating in costly *in vivo* trials.

### 5.6 Future perspectives

The next frontier in metal-targeted neuroprotection must go beyond traditional single-target pharmacology and integrate computational systems biology with precise nanomedicine. Given the extreme biochemical complexity involved in regulating unstable metal pools, predicting the off-target transcriptional toxicity of novel interventions remains a formidable challenge. Future breakthroughs will heavily rely on the combination of single-cell RNA sequencing (scRNA-seq) and gene perturbation computational models (*in silico* virtual knockouts). This “digital twin” prediction paradigm enables researchers to precisely simulate how novel targeted nanomedicines dynamically reconfigure the gene regulatory networks of specific glial cells and neuronal subpopulations before costly *in vivo* experiments, thereby establishing strict safety boundaries at the transcriptomic level (Kamimoto et al., 2023).

Furthermore, optimizing non-invasive drug delivery routes and intelligent delivery carriers represents a crucial development trajectory. The combination of the ‘nose-to-brain’ delivery route with multifunctional nanocarriers—like ROS-responsive polymer backbones grafted with mitochondrial-targeting peptides (such as SS-31)—is expected to effectively bypass the BBB and overcome systemic clearance (Lochhead and Thorne, 2012; Saravanakumar et al., 2017). This highly compartmentalized delivery strategy is designed to undergo specific cleavage and release metal chelators or nanoenzymes only within the oxidative microenvironment of ischemic or hemorrhagic penumbra. This will maximize the local subcellular bioavailability while avoiding systemic metal interference. In the end, combining *in silico* network inference with the multidisciplinary crossover of environment-responsive nanomedicine will provide a decisive pharmacological roadmap for successfully translating targeted bio-metal therapy into clinical practice.

## 6 Conclusion

In summary, maintaining the homeostasis of metal ions represents a highly promising—yet biochemically complex—therapeutic frontier in the treatment of neurological disorders. However, this review has certain limitations. We did not clearly define the exact regulatory effects of specific metal ions on neurological diseases. While this review primarily focuses on the metal ions most profoundly implicated in CNS pathologies (e.g., Fe, Cu, Zn, and Mn), the broader metallomic landscape may harbor additional undiscovered regulatory networks. In conclusion, on the basis of the clear demonstration of therapeutic potential of targeted metal neuroprotective intervention measures (such as smart chelators and targeted nanocarriers), future research should focus on complex metal ion regulatory networks and the permeability of the BBB. Introducing computational modeling of gene perturbations (*in silico*) could help evaluate these interventions, thereby paving the way for the successful transition of targeted metal therapies into clinical practice.

## Glossary

ABI: Acquired Brain Injury
AD: Alzheimer’s Disease
AIS: Acute Ischemic Stroke
ALOX15: Arachidonic Acid 15-Lipoxygenase
ALP: Autophagy-Lysosome Pathway
ALS: Amyotrophic Lateral Sclerosis
AMPAR: α-amino-3-hydroxy-5-methyl-4-isoxazolepropionic Acid Receptor
APP: Amyloid Precursor Protein
ATP: Adenosine Triphosphate
ATP7A/B: Copper Transport ATPase A/B
Aβ: Amyloid-beta
BACE1: β-site Amyloid Precursor Protein Cleaving Enzyme 1
BBB: Blood-Brain Barrier
BDNF: Brain-Derived Neurotrophic Factor
BPAN: β-propeller Protein-Associated Neurodegeneration
CAG: Cytosine-Adenine-Guanine
CaNa2EDTA: Calcium Disodium Ethylenediamine Tetraacetate
CcO: Cytochrome c Oxidase
CCS: Copper Chaperone for Superoxide Dismutase (Copper partner)
CNS: Central Nervous System
CO: Carbon Monoxide
Cp: Ceruloplasmin
Cr: Chromium
Ctr1: Copper Transporter 1
Cu: Copper
CuZn-SOD: Copper-Zinc Superoxide Dismutase
CVA: Cerebrovascular Accident
DFO: Deferoxamine
DMT1: Divalent Metal Transporter 1
ER: Endoplasmic Reticulum
FA: Friedreich’s Ataxia
fALS: Familial Amyotrophic Lateral Sclerosis
Fe: Iron
GCase: Glucocerebrosidase
GP: Globus Pallidus
GPX4: Glutathione Peroxidase 4
GSH: Glutathione
HD: Huntington’s Disease
HHT: Hereditary Hemorrhagic Telangiectasia
HO-1: Heme Oxygenase-1
HTT: Huntingtin Gene
ICH: Intracerebral Hemorrhage
ICP: Intracranial Pressure
IMM: Inner Mitochondrial Membrane
LCPs: Labile Copper Pools
LIP: Labile Iron Pool
LogP: Partition Coefficient
MAPK: Mitogen-Activated Protein Kinase
MAPT: Microtubule-Associated Protein Tau
MDS-UPDRS: Movement Disorder Society-Sponsored Revision of the Unified Parkinson’s Disease Rating Scale
mHTT: Mutant Huntingtin Protein
Mn: Manganese
Mn-SOD: Manganese Superoxide Dismutase
MPAC: Metal Protein-Attenuating Compound
MPTP: 1-methyl-4-phenyl-1,2,3,6-tetrahydropyridine (PD modeling agent)
MT: Metallothionein
MW: Molecular Weight
NADPH: Nicotinamide Adenine Dinucleotide Phosphate
nCp-Cu: Non-Ceruloplasmin Bound Copper (Free Copper)
NFT: Neurofibrillary Tangles
NMDAR: N-methyl-D-aspartate Receptor
NSCs: Neural Stem Cells
OPCs: Oligodendrocyte Precursor Cells
P2X7: P2X7 Purinergic Receptors
PARP-1: Poly(ADP-ribose) Polymerase 1
PD: Parkinson’s Disease
PET/CT: Positron Emission Tomography/Computed Tomography
PKAN: Pantothenate Kinase-Associated Neurodegeneration
PPB: Plasma Protein Binding
preHD: Premanifest Huntington’s Disease
ROS: Reactive Oxygen Species
sALS: Sporadic Amyotrophic Lateral Sclerosis
Se: Selenium
SNpc: Substantia Nigra pars compacta
SOD1: Superoxide Dismutase 1
Sp1: Specific Protein 1
TBI: Traumatic Brain Injury
TCA: Tricarboxylic Acid
TETA: Triethylenetetramine
TFAP2c: Transcription Factor AP-2c
TfR/TFR1: Transferrin Receptor 1
TTM: Tetrathiomolybdate
VD: Vascular Dementia
WD: Wilson’s Disease
Zn: Zinc
ZnO-NPs: Zinc Oxide Nanoparticles
